# Therapeutic Targets and Approaches to Manage Inflammation of NAFLD

**DOI:** 10.3390/biomedicines13020393

**Published:** 2025-02-06

**Authors:** Wanying Geng, Wanying Liao, Xinyuan Cao, Yingyun Yang

**Affiliations:** 1 4+4 Medical Doctor Program, Chinese Academy of Medical Sciences, Peking Union Medical College, Beijing 100730, China; gwy_0305@163.com; 2 Department of Gastroenterology, Department of Internal Medicine, Peking Union Medical College Hospital, Chinese Academy of Medical Sciences, Peking Union Medical College, Beijing 100730, China; liaowy17@mails.tsinghua.edu.cn (W.L.); caoxypumc@163.com (X.C.)

**Keywords:** NAFLD, NASH, inflammation, pathophysiology, therapy

## Abstract

Non-alcoholic fatty liver disease (NAFLD) and its advanced form, non-alcoholic steatohepatitis (NASH), are the leading causes of chronic liver disease globally. They are driven by complex mechanisms where inflammation plays a pivotal role in disease progression. Current therapies, including lifestyle changes and pharmacological agents, are limited in efficacy, particularly in addressing the advanced stages of the disease. Emerging approaches targeting inflammation, metabolic dysfunction, and fibrosis offer promising new directions, though challenges such as treatment complexity and heterogeneity persist. This review concludes the main therapeutic targets and approaches to manage inflammation currently and emphasizes the critical need for future drug development and combination therapy for NAFLD/NASH management.

## 1. Introduction

Non-alcoholic fatty liver disease (NAFLD) has emerged as the most common chronic liver disease, with prevalence rates estimated between 23% and 38% depending on the geographical region [1,2]. NAFLD encompasses a wide spectrum of liver conditions, ranging from non-alcoholic fatty liver (NAFL) to non-alcoholic steatohepatitis (NASH) [3]. NAFL is considered as a reversible condition, which involves relatively simple steatosis without significant inflammation. In contrast, NASH is marked by hepatocyte ballooning degeneration, diffuse lobular inflammation, and a higher risk of progressing to fibrosis, cirrhosis, and hepatocellular carcinoma (HCC) [4]. The definitive method for diagnosing NASH and distinguishing it from NAFL remains liver biopsy. Steatosis activity fibrosis (SAF) score and the NAFLD activity score (NAS) are the key assessment criteria for staging NASH, with NAS being the more commonly utilized system in clinical practice [5].

**Figure 1:**
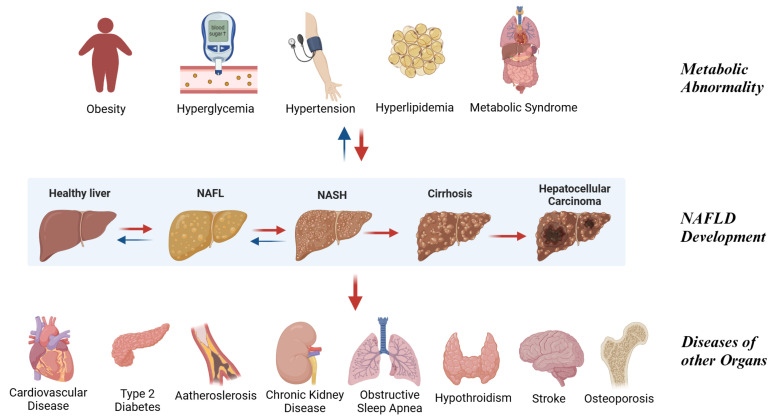
Non-alcoholic fatty liver disease (NAFLD) as an important driving force of multisystemic disorders. NAFLD is a critical contributor to multisystemic disorders. It progresses from simple steatosis (NAFL) to non-alcoholic steatohepatitis (NASH), cirrhosis, and hepatocellular carcinoma, significantly impacting liver health. Metabolic abnormalities such as obesity, hyperglycemia, hypertension, and hyperlipidemia closely interact with NAFLD. Additionally, NAFLD is linked to systemic complications, including cardiovascular disease, type 2 diabetes, chronic kidney disease, obstructive sleep apnea, and osteoporosis, emphasizing its role as a multifaceted metabolic condition. (Created in https://BioRender.com).

In the early stages of NAFLD, liver symptoms are often not obvious, and cardiovascular events are the main cause of death in NAFLD. In the later stages, liver-related causes of death such as decompensated cirrhosis or HCC dominate [6]. As a metabolic-related disease, NAFLD is often associated with other conditions, including obesity, type 2 diabetes, hyperlipidemia, hypertension, and metabolic syndrome (Figure 1). Obesity and insulin resistance (IR) contribute to disrupted lipid metabolism and persistent inflammation, driving the progression of NAFL to NASH, and potentially advancing to cirrhosis, HCC, and increased mortality [7]. Because of its close relation with metabolism, a recent consensus has proposed renaming NAFLD to metabolic dysfunction-associated steatotic liver disease (MASLD) [8]. However, as most clinical studies still use NAFLD as the primary term, this review retains the traditional nomenclature while acknowledging the ongoing debate about the terminology.

Due to its high prevalence and significant impact on global health, NAFLD has become a key focus for researchers. However, despite steady progress in epidemiology, pathophysiology, and potential therapeutic targets, significant gaps remain in the treatment of the disease. Some drugs have been terminated from clinical trials due to poor efficacy or adverse effects, while some of the others are currently undergoing clinical trials, but no drugs have been approved by authoritative institutions to date [9]. The development of NASH medications faces numerous challenges, closely tied to its complex pathogenesis—the “multiple hit” theory, in which inflammation plays a critical role in disease progression [10]. Therefore, a deeper understanding of the inflammatory signaling pathways and related mechanisms, along with identifying effective targets for specific inflammatory processes, will provide more insights and new strategies for treating NAFLD.

This review aims to provide an overview of the pathophysiology mechanisms related to inflammation in the occurrence and development of NAFLD/NASH, and also highlight the current progress, challenges, and potential future strategies for managing inflammation in NAFLD.

## 2. Pathophysiology of NAFLD-Related Inflammation

### 2.1. Adipocyte Death

Adipose tissue is the largest endocrine organ that secretes several pro-inflammatory cytokines and adipokines, including leptin, Tumor Necrosis Factor α (TNF-α), and interleukin (IL)-6, which affect liver inflammation and insulin resistance [11,12]. The common pattern of altered circulating adipokines in NAFLD often includes elevated leptin levels and reduced adiponectin levels, both of which are linked to disease severity [12]. Leptin can contribute to inflammation by activating Kupffer cells (KCs) and stimulating them to secrete TNFα [13]. However, some studies suggest that leptin seems to have bidirectional action in the development of NASH [14]. In contrast, adiponectin has a protective role, as it inhibits hepatic stellate cell (HSC) proliferation [15] and decreases the TNF-α production in macrophages [16]. Meanwhile, adiponectin can also promote KCs to polarize into the M2 phenotype, stimulating the production of anti-inflammatory cytokines like IL-10 [17]. In addition, previous studies have shown that adiponectin can reduce IR and liver steatosis, with additional anti-inflammatory, anti-apoptotic, and insulin-sensitizing properties, whereas TNF increases IR and has pro-inflammatory effects [18]. Elevated TNF levels, alongside low adiponectin levels, are linked to IR and drive NAFLD progression, even in healthy individuals [19]. Moreover, some lipotoxic substances, such as ceramides and palmitic acid, are released by adipocytes during lipolysis. They can result in cellular stress responses such as ER stress and oxidative stress, which are known to drive hepatocyte apoptosis and necrosis [20].

Finally, adipocyte death increases the levels of circulating free fatty acids, further promoting lipotoxicity in the liver. Obesity exacerbates this process by increasing adipocyte death, leading to the recruitment of chemokine C-C motif receptor 2 (CCR2)^+^ macrophages, which play a key role in liver injury and fibrosis in NASH [21]. Thus, adipocyte death and its consequences, including increased macrophage activation and lipid overload, are key drivers of the inflammatory and fibrotic processes in NAFLD.

### 2.2. Hepatocyte Death

Hepatocyte death is one of the central events in NASH development because it triggers liver inflammation [22]. Different modes of cell death, including apoptosis, necrosis, necroptosis, and pyroptosis, play different roles in the progression of liver disease. Each pathway activates unique mechanisms that initiate cell death responses, such as the release of damage-associated molecular patterns (DAMPs) from cells [23,24,25].

Hepatocyte apoptosis is considered as the key driving factor regarding hepatocyte death in NASH [26]. It is markedly elevated in both human and animal models of NASH, correlating positively with disease severity and serving as a key pathological hallmark of the condition [27]. Immunohistochemical assays demonstrated positive results for hepatocyte caspase-3 cleavage and TUNEL positivity in liver tissues from patients and mouse models with NASH. AMP-activated protein kinase (AMPK) phosphorylates the pro-apoptotic protein caspase-6 to inhibit its activation, thereby controlling hepatocyte apoptosis. In NASH, AMPK activity is suppressed. The activation of AMPK or inhibition of caspase-6, even after the onset of NASH, can improve liver injury and fibrosis [28]. Lei et al. also identified a novel anti-apoptotic CD1d-JAK2-STAT3 axis in hepatocytes, which provides hepatoprotective effects. This discovery underscores the potential of targeting hepatocyte CD1d as a therapeutic strategy for managing liver injury and fibrosis in NASH [29].

However, increasing evidence suggests that the role of apoptosis in the inflammation and progression of NASH may be overestimated, as other types of hepatocyte death also make great contribution [26]. Necrosis and necroinflammation are characteristic histological features of NASH. In patients with NASH, the expression of hepatic *RIPK3*, a marker of necroptosis, was significantly upregulated, while the level of intrahepatic caspase-3 cleavage was lower than in healthy controls. The results indicated a shift in liver cell death signaling from apoptosis towards necroptosis. Hepatocyte autophagy is impaired in patients with NAFLD [30]. Rubicon, a Beclin1-interacting negative regulator for autophagosome-lysosome fusion, is significantly elevated in the liver tissue of NAFLD patients. The overexpression of Rubicon contributes to pathogenesis by promoting hepatocellular lipoapoptosis, lipid accumulation, and autophagy inhibition [31].

Recent findings indicate that pyroptosis also plays a critical role in liver inflammation. This process relies on the inflammasome-mediated activation of caspase-1, which triggers Gasdermin D (GSDMD) insertion into the plasma membrane, forming pores that lead to the release of intracellular proteins, ion imbalance, water influx, and cell swelling [32]. Yun Feng et. [33] found that p-STAT3 enhances *Anxa2* expression, activating caspase-1 and thereby promoting hepatocyte pyroptosis and fibrosis in NASH. Inhibiting p-STAT3 or caspase-1 can effectively reduce disease progression. Another study shows that SMS1 mediates hepatocyte pyroptosis via the DAG-PKCδ-NLRC4 axis, and inhibiting SMS1 or related pathways can prevent the progression of NASH [34].

### 2.3. Oxidative Stress and Endoplasmic Reticulum Stress

Oxidative stress arises from the excessive production of reactive oxygen species (ROS), which are natural byproducts of aerobic metabolism. In NAFLD, elevated ROS levels exacerbate hepatic fibrosis by activating HSCs to produce extracellular matrix components [35]. Furthermore, ROS act as critical regulators of NF-κB activity, promoting immune and inflammatory pathways that aggravate liver damage [36]. Oxidative stress also generates oxidized phospholipids (OxPLs), which impair mitochondrial function in hepatocytes, leading to inflammation and fibrosis. Studies have shown that neutralizing OxPL alleviates oxidative stress and its downstream effects [37].

Additionally, sustained ROS production causes endoplasmic reticulum (ER) stress and mitochondrial dysfunction, with the latter marked by electron transport chain (ETC) damage, reduced ATP synthesis, and lipid peroxidation [38]. ER stress is a cellular response triggered to restore protein homeostasis through the unfolded protein response (UPR). However, unresolved ER stress activates pro-apoptotic pathways, leading to cell death and the exacerbation of hepatic steatosis. The UPR involves three key ER-resident stress sensors: PERK, IRE1, and ATF6, which are normally bound to glucose-regulated protein 78 (GRP78) in an inactive state [39,40]. Prolonged ER stress promotes adipogenesis via SREBP-1c activation and disrupts lipid homeostasis by increasing lipid input and decreasing lipid output pathways. Moreover, ER stress-induced apoptosis is mediated through the PERK-eIF2α-ATF4, IRE1-XBP1, and ATF6-CHOP signaling pathways, which collectively amplify inflammation and cell death [41]. These mechanisms underscore the critical roles of oxidative and ER stress in driving the transition of NAFLD to more severe forms, such as NASH.

### 2.4. Gut Microbiome

The gut microbiome plays a crucial role in the pathogenesis of NAFLD through its effects on the gut–liver axis. Research has shown that patients with advanced NAFLD and NASH often have specific changes in their gut microbiota. For instance, *Escherichia coli* and *Bacteroides vulgatus* are more abundant in patients with advanced fibrosis [42]. Furthermore, alterations in the gut barrier, including a reduction in tight junction proteins like zonula occludens-1 and occludin, lead to increased gut permeability, allowing bacterial products such as lipopolysaccharide (LPS) to translocate from the gut into the portal circulation [43]. When it reaches the liver, LPS activates Toll-like receptors (TLRs) on hepatic cells, triggering an inflammatory response that exacerbates liver injury [44]. In addition, the development of hepatitis and NASH was prevented after the administration of local intestinal antibiotics, highlighting the significant role of the microbiota in promoting liver inflammation in NASH [45].

The bile acid cycle is another critical component of the gut–liver axis. Bile acids (BAs) regulate lipid metabolism and inflammation through receptors such as farnesoid X receptor (FXR) and G protein-coupled BA receptor 1 (TGR5). Moreover, BAs and the gut microbiota interact in a reciprocal manner: BAs influence the intestinal microbiome through direct antimicrobial effects and the FXR-induced production of antimicrobial peptides, while the gut microbiota alters the BA pool composition through specific enzymatic processes, including deconjugation, oxidation, epimerization, dehydroxylation, etc. [46]. In NAFLD/NASH, dysregulated bile acid metabolism promotes hepatic steatosis and fibrosis [47,48]. Experimental evidence has shown that targeting bile acid pathways with FXR and TGR5 agonists, such as obeticholic acid, can reduce hepatic steatosis and inflammation, highlighting the potential of gut–liver axis therapies in managing NAFLD/NASH.

### 2.5. Dietary Habits

Diet is a significant modifiable factor in the development and progression of NAFLD/NASH, with unhealthy dietary habits contributing to both liver inflammation and metabolic dysfunction. The Western diet, characterized by low choline and high levels of sugar, fat, and processed foods, leads to the production of 2-oleoylglycerol in Western-diet-fed mice, contributing to liver inflammation and fibrosis [49].

High-fructose intake, in particular, has been linked to increased intestinal permeability and endotoxin translocation from the gut to the liver [50]. Fructose bypasses key regulatory pathways in glucose metabolism, leading to de novo lipogenesis (DNL) in the liver, which results in an accumulation of triglycerides and other lipotoxic lipids [51]. Fructose also promotes the upregulation of inflammatory genes in the liver and triggers mitochondrial dysfunction, exacerbating hepatic oxidative stress and inflammation [52]. Moreover, high-starch carbohydrates can aggravate NAFLD by promoting fatty acid influx and upregulating the intrahepatic expression of NADPH oxidase 2 (NOX2) [53]. Meanwhile, excessive intake of saturated fatty acids and cholesterol in the diet can also lead to the development of NASH. Research has shown that increased intake of saturated fatty acids in the diet can lead to unfolded protein response (UPR), which in turn causes ER stress and cell apoptosis [54]. The association between cholesterol and NASH severity has also been confirmed in mouse models. Mice fed with a high-cholesterol diet exhibit strong inflammatory responses in their livers [55]. In addition, free cholesterol can also accumulate directly in KCs and HSCs, activating liver inflammation and fibrosis [56].

Thus, diet plays a critical role in modulating the inflammatory processes in NAFLD/NASH, with high-fructose, high-fat/high-cholesterol diets promoting liver injury, while healthier dietary patterns can alleviate inflammation and metabolic dysfunction [44,57].

## 3. Inflammatory Targets for NASH

### 3.1. Non-Pharmacological Therapy

#### 3.1.1. Lifestyle Modification

Lifestyle modification, including a healthy diet and regular exercise, is the foundation of NAFLD/NASH treatment. It helps reduce metabolic overload, improves adipose tissue function, and alleviates metabolic-inflammatory stress [58].

Foods high in saturated fats, cholesterol, refined carbohydrates, sugary drinks, red meat, and highly processed items are strongly linked to the development of NAFLD/NASH. In contrast, a calorie-restricted diet, like the Mediterranean diet, can reduce total body fat, visceral fat, and intrahepatic lipid content [59]. Research showed that gradual weight loss, achieved by reducing daily calorie intake by 500–1000 kcal, can enhance insulin sensitivity, and has other potential metabolic, cardiovascular, and liver health benefits [60]. Other dietary components that may be beneficial for NAFLD include omega-3 polyunsaturated fatty acids (PUFAs) and coffee. Growing evidence suggests that omega-3 PUFAs have therapeutic benefits for metabolic diseases due to their antioxidant and anti-inflammatory effects [61]. A study using proteomic methods found that supplementing n-3 PUFA improved adipogenic markers, ER stress, and mitochondrial function in NASH patients [62]. In addition, a recent meta-analysis involving 22 RCTs with 1366 participants reported a decrease in liver fat and a significant improvement in the levels of triglyceride, total cholesterol, high-density lipoprotein, and body mass index with PUFA treatment [63]. Besides omega-3 PUFAs, research showed that coffee consumption had an inverse relationship with adverse liver health outcomes by reducing aminotransferase levels, slowing fibrosis progression [64], and even lowering liver-related mortality [65]. Regular coffee consumption has consistently been linked to a lower risk of NAFLD, and observational studies suggest the association between them may be in a dose–response way [66]. However, there is still controversy over whether n-3 PUFA and alcohol can delay disease progression and achieve treatment goals [61].

Exercise is another effective way to manage NAFLD/NASH. Exercise training intervention is considered to help reduce intrahepatic fat accumulation, lower blood pressure, and improve insulin resistance in obese people [67]. Specifically, both aerobic and resistance exercises are beneficial [68], and exercise intensity may be more critical than duration or total exercise volume [69]. In NASH patients, long-term exercise can enhance the phagocytic ability of KCs in the liver, thereby reducing liver inflammation and preventing disease progression [70]. A study in Korea also showed that exercise can induce irisin which is competitively bound with MD2, disrupting the formation of the MD2-TLR4 complex. This process inhibited downstream inflammatory response and alleviated hepatic steatosis and fibrosis in NASH [71].

#### 3.1.2. Bariatric Surgery

As a long-term weight loss method, weight loss surgery can reduce food absorption, improve metabolic disorders, and thereby alleviate obesity-related diseases [72]. At present, the most common bariatric surgery are laparoscopic sleeve gastrectomy (LSG) and Roux-en-Y gastric bypass surgery [73]. The current guidelines suggest that bariatric surgery can be used to treat NAFLD and improve the cardiac metabolic health of NAFLD patients with Body Mass Index (BMI) > 35 kg/m^2^ (>32.5 kg/m^2^ in the Asian population), especially those with type 2 diabetes. For individuals with a BMI > 30–34.9 kg/m^2^ (>27.5–32.4 kg/m^2^ in Asian population), weight loss surgery should also be considered [74]. A recent meta-analysis included 37 studies indicating that bariatric surgery alleviates liver steatosis, inflammation, and fibrosis in NASH patients, and also significantly reduces the average NAS [3]. In terms of surgical methods, RYGB achieved the most significant improvement in steatosis, while LSG in fibrosis [75]. In addition, bariatric surgery can also reduce the progression of NAFLD to cirrhosis [76], as well as lower the risk of cancer [77] and adverse cardiovascular events in NAFLD patients with severe obesity [78]. However, it is worth noting that there is some risk of developing new or worsened NAFLD after bariatric surgery [79].

#### 3.1.3. Modification of Gut Microbiota

Gut microbial dysbiosis is considered as a pathogenic mechanism of NASH, and the types and abundance of microbiota are correlated with the progression of the disease. Various strategies for modulating gut microbiota, such as fecal microbiota transplantation (FMT), pharmacological approaches (e.g., antibiotics and probiotics), and others, have shown potential for improving liver disease outcomes [80]. FMT is the infusion of feces from a healthy donor into the gastrointestinal tract of a recipient patient to treat diseases associated with gut microbiota imbalances [81]. Zhou et al. found that after eight weeks of FMT treatment, the gut microbiota disorder in NASH mice fed with HFD was corrected. At the same time, steatohepatitis was relieved after FMT, manifested by a decrease in hepatic lipid accumulation, hepatic pro-inflammatory cytokines, and NAS [82]. Another randomized control study showed that an FMT from a thin and healthy donor to NAFLD patients reduced intestinal permeability, but could not improve insulin resistance and hepatic proton density fat fraction [83].

Probiotics colonize the host’s gut to alter the composition of the gut microbiota, which helps maintain intestinal barrier integrity, reduce inflammatory factors, and prevent bacterial translocation [84]. Al-Muzafar et al. reported that a probiotic mixture helps alleviate HFSD-induced steatosis in mice by influencing leptin, resistin, and inflammatory biomarkers, and markers of liver function [85]. Similarly, another research indicated probiotics could improve liver pathology, and also delay the progression of NAFLD by downregulating serum LPS and liver TLR4 [86]. In clinical practice, similar effects have also been observed with the use of probiotics. In a randomized, double-blinded study, researchers found that taking a high-potency multistrain probiotic for one year significantly reduced alanine aminotransferase and cytokine levels in NAFLD patients, and improved hepatocyte ballooning (*p* = 0.036), lobular inflammation (*p* = 0.003), and NAS (*p* = 0.007) [87]. However, in another study, probiotics did not lead to significant clinical improvements, but they appeared to help stabilize mucosal immune function and protect against increased intestinal permeability in NAFLD patients [88]. A separate study suggested that a novel treatment combining capsaicin with antibiotics reduced hepatic fat accumulation, obesity, and metabolic disorders [89]. However, the potential risks of antibiotics must be thoroughly evaluated before use in human patients.

In the future, additional studies with larger sample sizes and extended durations are necessary to accurately assess the potential benefits of modification of the gut microbiota in NAFLD.

#### 3.1.4. Liver Transplantation

Liver transplantation is the only life-saving option for patients with NASH-related end-stage liver disease (ESLD) and unresectable HCC [90]. Over the past decade, NAFLD, particularly NASH-related cirrhosis, has become the fastest-growing indication for liver transplantation in Western countries [91]. A report in the United States indicated that NAFLD, with or without HCC, is now the most common indication for liver transplantation (LT) in women and the second most common in men [92]. Multiple studies have shown that the overall mortality after liver transplantation in patients with NASH-related cirrhosis is associated with factors such as age, obesity, type 2 diabetes, and post-transplant metabolic syndrome [93,94,95]. Therefore, the careful assessment and appropriate management of metabolic comorbidities before and after transplantation are essential, as these factors may impact eligibility for liver transplantation and post-transplant outcomes. Additionally, lifestyle modifications, immune-suppressant management, and specific pharmacologic treatments for coexisting cardiometabolic complications are the cornerstone of post-transplant care [91]. Prospective longitudinal studies are needed in the future to optimize treatment outcomes for NAFLD/NASH patients undergoing liver transplantation.

### 3.2. Natural Compounds

#### 3.2.1. Vitamin E

Vitamin E is a fat-soluble vitamin with eight natural forms, including four tocopherols and four tocotrienols [96]. It is an important antioxidant in the human body, exerting biological activities such as antioxidant, anti-inflammatory, and anti-apoptotic, especially in clearing free radicals and blocking lipid oxidation [97]. In NASH patients, the plasma levels of vitamin E (α-tocopherol) are reduced [98]. Studies have also shown that vitamin E inhibited hepatic TGF-β expression, which helped to lessen steatosis, inflammation, and fibrosis in patients with NAFLD, which indicated that Vitamin E is a potent antioxidant with promising potential for NAFLD treatment [99]. Research in animal NASH models demonstrated that vitamin E had positive effects on NAFLD through several mechanisms, including the enhancement of lipid and glucose metabolism via the Nrf2/CES1 pathway activation [100] and a reduction in oxidative stress through the inhibition of iNOS and NADPH oxidase [101]. The PIVENS trial, a 96-week study with 247 NASH patients receiving either 800 IU/day of vitamin E or a placebo, found significant improvements in liver enzyme levels and histological features in the vitamin E-receiving group [102]. The 2020 Guidelines in Japan stated that vitamin E can be used to treat NASH [103]. Other studies showed that in NASH patients with or without diabetes, vitamin E can improve steatosis, liver inflammation, and ballooning, but cannot improve fibrosis [103,104]. A randomized controlled trial in China demonstrated that diabetic patients with the haptoglobin (Hp) 2–2 allele responded more effectively to vitamin E therapy. Notably, the prevalence of the Hp 2–2 allele in Chinese NAFL/NASH patients is higher than in Western patients, suggesting that Chinese patients may derive greater benefit from vitamin E treatment [105]. A recent research, planning to evaluate the minimal effective dose of vitamin E, concluded that the clinical effect was independent of dose. Additionally, vitamin E may be sequestered in lipid droplets, making it challenging to determine its appropriate dose [106]. However, in the long term, vitamin E may be associated with some potential risks such as prostate cancer, stroke, and mortality [107], so further studies should focus more on its action mechanism, optimal dose, and safety of long-term use.

#### 3.2.2. Other Natural Compounds

In addition to vitamin E, other natural compounds have been evaluated for their effectiveness in NASH treatment. Silymarin has shown varying effects in clinical trials for NASH treatment. A double-blind trial in 2017 demonstrated fibrosis reduction in many patients and AST/platelet ratio improvement [108]. However, a recent phase II multicenter trial indicated no significant changes in liver steatosis or biochemical markers (ALT and AST), and gastrointestinal side effects affected 56% of the participants [109]. Another option is resveratrol, which is considered to have antioxidant, anti-inflammatory, anticancer, and cardiovascular protective effects in in vitro and animal experiments. A study showed significant benefits on AST, anti-inflammatory cytokines, and steatosis in NAFLD patients, surpassing the effects of lifestyle changes [110]. However, a separate trial using higher doses found no impact on hepatic lipid content, likely due to the small sample size and dose limitation [111]. Curcumin is extracted from the rhizomes of some ginger plants and is believed to have antioxidant, liver protection, and antifibrotic effects. However, many trials showed minimal efficacy in fibrosis reduction or metabolic parameters compared to placebo [112,113]. Lastly, caffeine, combined with chlorogenic acid, showed modest benefits in T2DM patients, with notable weight loss and an increase in beneficial Bifidobacterium species [114]. Another trial reported reduced cholesterol in the caffeine group and increased insulin with the caffeine–chlorogenic acid combination, but with no liver-specific outcomes [115].

### 3.3. Pharmacotherapies

**Figure 2:**
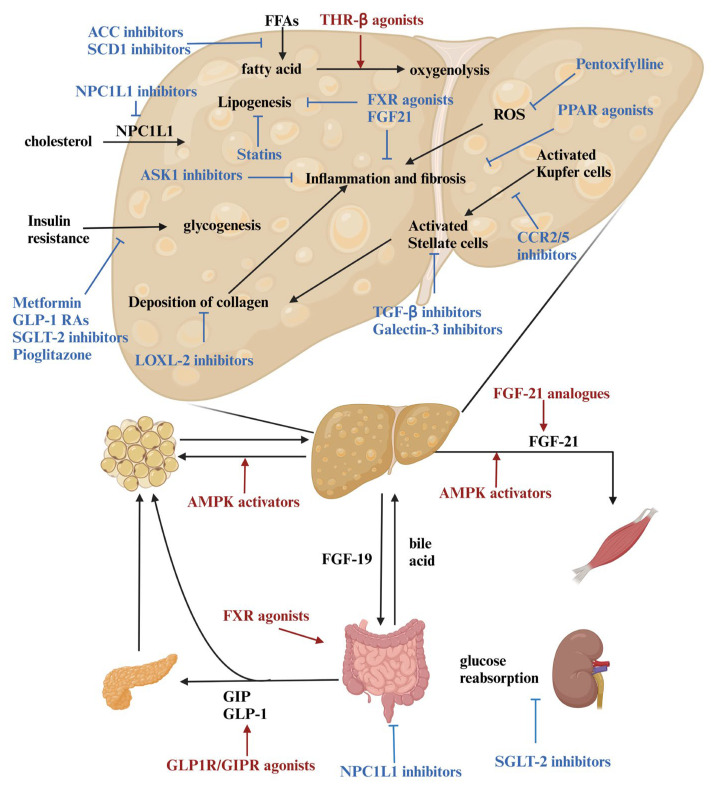
Drug targets for the treatment of NAFLD/NASH. This figure illustrates key drug targets and mechanisms for the treatment of NAFLD/NASH. It highlights the pathways involved in metabolic regulation, anti-inflammatory strategies, bile acid metabolism modulation, and antifibrotic approaches. These targets address critical pathological processes, including metabolic dysregulation, inflammation, and liver fibrosis, providing a framework for precision therapy in NAFLD/NASH. (Created in https://BioRender.com). Black words indicate the pathway or molecules acting on that pathway; The red and blue words contain the names of drug targets. The red words and lines together indicate that the drug target activates the pathway, while the blue words and lines together indicate that the drug target exerts an inhibitory effect.

Despite significant advancements in understanding the pathological mechanisms of NAFLD, no FDA- or EMA-approved treatments are available due to side effects or insufficient clinical efficacy. However, a growing pipeline of novel drugs offers promising potential for addressing NASH. The current clinical trials focus on two primary goals: NASH resolution without worsening fibrosis or fibrosis improvement without worsening NASH. Therapeutic approaches can be broadly categorized into monotherapies addressing the metabolic, anti-inflammatory, or antifibrotic pathways, and combination therapies designed to enhance efficacy and mitigate adverse effects. This review summarizes key targets and the latest progress in NASH drug development (Figure 2).

#### 3.3.1. Targeting Glucose and Lipid Metabolisms

##### AMPK Activators

Metformin, an indirect activator of AMPK, effectively reduces glucose production in hepatocytes and improves insulin resistance, which is widely recommended as first-line therapy for T2DM [116]. In terms of liver disease, metformin has shown diverse results. Despite its ability to improve liver enzymes and HbA1c in T2DM patients with NAFLD, randomized controlled trials (RCTs) have demonstrated limited impact on liver histology in NAFLD and no significant effects on NASH resolution or fibrosis [117,118]. Consequently, major guidelines, including EASL-EASO-EASD, AASLD, and NICE, do not recommend metformin for treating NAFLD/NASH [92,119,120].

However, metformin may offer protective effects in chronic liver disease, particularly by reducing the risk of cirrhosis and HCC [121,122,123]. Retrospective studies and meta-analyses have highlighted its association with reduced HCC incidence and improved survival in T2DM patients with chronic liver disease or HCC. For instance, long-term metformin use in T2DM patients with biopsy-proven NASH and advanced fibrosis/cirrhosis was linked to lower all-cause mortality and HCC incidence over seven years [122]. Although observational evidence suggests potential anticancer benefits, further RCTs are necessary to confirm these findings.

##### SGLT-2 Inhibitors

SGLT2 inhibitors, a novel class of antidiabetic drugs, reduce glucose reabsorption in the renal proximal tubules [124]. Beyond their glycemic effects, these agents show promise in improving liver health, particularly in patients with NAFL/NASH and T2DM, by reducing steatosis, fibrosis, and inflammation through mechanisms such as inhibiting KC activation [125,126].

Clinical trials have highlighted specific benefits of SGLT2 inhibitors on liver function. The long-term use of ipragliflozin (50 mg/day) significantly improved liver fibrosis, function, and steatosis, with 67% of the treated patients achieving NASH resolution compared to 27.3% in controls, and no new NASH cases reported in the treatment group [127,128]. Similarly, empagliflozin (25 mg/day for 24 weeks) reduced steatosis, ballooning, and fibrosis, alongside improvements in controlled attenuation parameter value and liver stiffness [129,130]. Canagliflozin demonstrated marked improvements in fibrosis markers and metabolic parameters, particularly in patients with fibrosis stages 1–3 [131]. Dapagliflozin also reduced visceral fat, liver enzymes, and fibrosis-related biomarkers in T2DM patients, though no benefit was observed in non-T2DM patients after 12 weeks of treatment [132,133].

However, it is important to note that most available RCTs on SGLT-2 inhibitors are now limited by small sample sizes and do not directly evaluate their effects on liver histology [134]. Therefore, larger and more detailed studies are needed to further explore their potential benefits.

##### PPAR Agonists

Peroxisome proliferator-activated receptors (PPARs), including three isoforms (PPAR-α, PPAR-γ, and PPAR-δ/β), are nuclear receptors that form heterodimers with retinoid X receptor (RXR) to regulate gene transcription [135]. These receptors are key regulators of lipid metabolism, glucose homeostasis, insulin resistance, inflammation, and fibrosis, with distinct tissue-specific roles in the liver, adipose tissue, muscle, and kidney. Given their central role in these processes, PPARs are promising therapeutic targets for NASH and other metabolic liver diseases [135].

Among the single agonists, PPARα agonists such as pemafibrate have shown improvements in liver stiffness and ALT levels in a phase II trial (NCT03350165), though they failed to meet the primary endpoints [136]. In addition, some preclinical studies with bezafibrate (GW7647) [137,138] and gemcabene [139] demonstrated antifibrotic effects and reduced hepatic inflammation in animal models. PPARγ agonists, like pioglitazone, significantly improved insulin sensitivity and achieved fibrosis regression in patients with T2DM during a phase IV trial (NCT00994682), though there were side effects such as weight gain and edema [140]. Seladelpar, a PPARδ ligand, showed the most success in PBC treatment. It was temporarily paused due to safety concerns in NASH, but subsequent evaluations cleared the drug for continued clinical trials [141]. However, results in NASH have been mixed, so further research is needed to be carried out.

Dual and pan-PPAR agonists combine the benefits of individual PPAR subtypes, providing superior outcomes in NASH treatment. Saroglitazar, a dual PPARα/γ agonist, demonstrated significant reductions in ALT levels (−44.3% at 4 mg) and liver fat content in a phase II trial (NCT03061721) and is currently under phase IV trials for metabolic comorbidities like obesity and T2DM (NCT05872269) [142]. Elafibranor, targeting PPARα/δ, resolved NASH without fibrosis worsening in a phase IIb trial (NCT01694849) [143], but it failed to achieve NASH resolution without worsening fibrosis in a phase III trial and was terminated [144]. Lanifibranor, a pan-PPAR agonist, achieved significant histological and metabolic benefits in a phase IIb trial (NCT03008070) [145], and is undergoing phase III evaluation with FDA breakthrough therapy designation. Other pan-PPAR agonists, such as chiglitazar, are in development, showing the potential for treating NASH [146]. Although dual and pan-PPAR agonists generally outperform single agonists, challenges like low metabolic stability and frequent side effects (e.g., diarrhea, nausea, and weight gain) persist, underscoring the need for optimization and combination strategies to maximize their therapeutic potential [147].

##### GLP1R/GCGR/GIPR Agonists

Two key incretins, glucagon-like peptide 1 (GLP1) and glucose-dependent insulinotropic polypeptide (GIP), play roles in glucose regulation, with GLP1 promoting insulin secretion and GIP modulating insulin or glucagon depending on blood glucose levels [148]. Given the metabolic overlap between T2DM and NAFLD, they become promising therapeutic options for NAFLD/NASH management.

Semaglutide, a GLP1R agonist, achieved the primary endpoint of NASH resolution without fibrosis worsening in a phase II trial (NCT02970942) and is now in phase III trials (NCT04822181) [149]. Efinopegdutide, a GLP1R and glucagon receptor (GCGR) dual agonist, significantly reduced liver fat content in a dose-dependent manner in a phase II trial (NCT04944992) and has received FDA fast-track designation for NASH [150]. Cotadutide also reduced liver lipid accumulation and improved inflammation and fibrosis in T2DM patients [151]. Another two GLP1R/GCGR dual agonists, Pemvidutide [152] and Survodutide [153], are under evaluation for NAFLD/NASH in phase I and II trials, respectively (NCT05006885 and NCT04771273). Tirzepatide, a GLP1R and GIP receptor (GIPR) dual agonist, reduced NASH biomarkers like ALT and AST in a phase II trial (NCT03131687) [154], with further studies ongoing (NCT04166773). Lastly, Retatrutide, a triple agonist of GCGR/GIPR/GLP1R, showed dose-dependent reductions in liver fat and high rates of NASH resolution phase IIa trial [155]. These results highlight the potential of incretin-based therapies to address liver fat, inflammation, and fibrosis in NAFLD/NASH.

##### HMG-CoA Reductase Inhibitors

Statins, HMG-CoA reductase inhibitors, restrict cholesterol synthesis and are primarily used as lipid-lowering medications. In the GREACE study, a post hoc analysis involving 437 NAFLD patients showed that statin treatment significantly reduced cardiovascular morbidity without major liver-related adverse events [156]. Additional evidence suggests that statins not only improve cardiovascular outcomes but also help alleviate liver conditions in NAFLD, such as steatosis, inflammation, and fibrosis. A large observational study in Korea found that statin use was associated with a reduced risk of NAFLD and a lower risk of liver fibrosis [157]. Furthermore, research showed Rosuvastatin can effectively control the cholesterol elevation caused by the FGF-19 analog NGM282 (Aldafermin), and their combined use may be a reasonable strategy to improve cardiovascular risk in NASH patients [158]. Another study used the Optum Clinformatics database to assess the risk of HCC in NAFLD patients initiating statin treatment. Statin users had a 53% lower risk of developing HCC, with greater reduction observed at higher doses and longer use. These findings suggest statins may reduce HCC risk in NAFLD patients [159]. Despite concerns about the hepatotoxic effects of statins, a post hoc analysis of statins indicated that statin therapy was safe for patients with prediabetes, T2DM, and NASH [160].

##### NPC1L1 Inhibitors

Ezetimibe is a lipid-lowering agent that reduces cholesterol absorption by inhibiting the NPC1L1 protein in intestinal epithelial cells and hepatocytes [161]. A meta-analysis of six studies with 273 NAFLD patients found that ezetimibe significantly reduced liver enzymes, and also improved hepatic steatosis and hepatocyte ballooning, but had no effect on liver fibrosis [162]. Meanwhile, ezetimibe is also effective and well tolerated in combination with other medicines, such as statins, orlistat, and insulin-sensitizing agents [163]. A study showed that the combination of ezetimibe and rosuvastatin significantly reduced liver fat compared to rosuvastatin alone (*p* = 0.020). Only the combination therapy improved CAP by transient elastography, while no significant changes in liver fibrosis were noted [164].

##### ACC Inhibitors

Acetyl-CoA carboxylase (ACC), conversing acetyl-CoA to malonyl-CoA, promotes de novo lipogenesis, inhibits mitochondrial fatty acid oxidation, and ultimately contributes to fat accumulation and steatosis. ACC inhibitors have shown promising potential in the treatment of NASH by targeting key metabolic pathways. In a randomized, placebo-controlled trial (NCT02856555), GS-0976, an ACC inhibitor, led to a 29% reduction in MRI-PDFF compared to 8% with placebo (*p* = 0.002), and also decreased hepatic DNL and improving fibrosis markers and liver biochemistry [165,166]. Similarly, MK-4074, a liver-specific ACC1/2 inhibitor, suppressed DNL and enhanced fatty acid oxidation (FAO) in preclinical and clinical studies, reducing hepatic triglycerides (TGs) by 36% in patients with steatosis but significantly increasing plasma TGs by 200% (NCT01431521) [167]. PF-05221304 demonstrated dose-dependent reductions in liver fat (50–65%) but also increased serum TG levels by 8%, which could be mitigated by combining it with the DGAT2 inhibitor PF-06865571 (NCT03248882 and NCT03776175) [168]. Additionally, NDI-010976, an allosteric ACC1/2 inhibitor, showed dose-dependent hepatic DNL inhibition and good tolerability in obese males (NCT02876796) [169].

##### SCD1 Inhibitors

Stearoyl-CoA desaturase 1 (SCD1) is a key enzyme that catalyzes the rate-limiting step in mono-unsaturated fatty acid (MUFA) synthesis, converting stearoyl-CoA and palmitoyl-CoA into oleate and palmitoleate [170]. Its expression is elevated in the livers of patients with NAFLD and ob/ob mice [171]. Targeting SCD1 through gene knockdown or inhibition has shown potential in reducing hepatic lipid accumulation and lipid toxicity in clinical trials [172]. Aramchol, a conjugate of cholic acid and arachidic acid, is a promising SCD1 inhibitor under investigation for NASH treatment. By inhibiting SCD1 and activating PPARγ, Aramchol reduces liver fat content and fibrogenic gene expression in human HSCs [173]. In a phase II trial (NCT01094158), treatment with 300 mg/day reduced liver fat by 12.57% compared to a 6.39% increase in the placebo group [174]. In a subsequent 52-week phase IIb trial (NCT02279524), 16.7% of the patients receiving 600 mg of Aramchol achieved NASH resolution without fibrosis worsening, versus 5% in the placebo group, while fibrosis improvement was observed in 29.5% compared to 17.5%, respectively [175]. Currently, it is being evaluated in a phase III trial (NCT04104321) to further assess its safety and efficacy.

##### THR-β Agonists

Thyroid hormone receptor beta (THR-β), representing 80% of the hepatic thyroxine receptors, plays a key role in cholesterol metabolism, bile acid excretion, fatty acid β-oxidation, and mitochondrial biogenesis [176]. By regulating these critical metabolic pathways, THR-β maintains liver homeostasis and function. In NASH, THR-β agonists have demonstrated the potential to reduce lipotoxicity, improve liver function, and decrease liver fat content [177,178]. Two THR-β agonists, resmetirom (MGL-3196) and VK2809, have shown promising results in phase 2 studies for NASH.

Resmetirom (MGL-3196), which is liver-directed and highly selective for THR-β, can not only reduce hepatic triglycerides, steatosis, inflammation, and fibrosis markers, but also minimize side effects on the heart and bones [177]. In a 36-week phase II trial (NCT02912260), resmetirom significantly reduced markers of fibrosis, including liver stiffness (*p* = 0.015) and PRO-C3/C3M ratio (*p* = 0.0004) [179]. The ongoing phase III trial (NCT03900429) is evaluating its efficacy at daily doses of 80 and 100 mg, with earlier studies demonstrating good safety and tolerability [179,180]. In March 2024, the U.S. Food and Drug Administration approved Rezdiffra (resmetirom) for the treatment of adults with noncirrhotic NASH with moderate to advanced liver fibrosis. This therapy is intended to be used in conjunction with diet and exercise to address the disease’s progression effectively.

VK2809, another liver-specific THR-β agonist, acts as a prodrug that releases active thyromimetics in vivo, improving lipid metabolism and cholesterol levels. In a phase II study (NCT02927184), VK2809 significantly reduced liver fat content, with a median reduction of 53.8% at 12 weeks and 88% of the patients achieving ≥30% fat reduction, without severe adverse effects reported [181]. On 4 June 2024, Viking Therapeutics reported that in a phase 2b trial, 69% of the patients treated with VK2809 achieved NASH resolution without fibrosis worsening compared to 29% in the placebo group (*p* < 0.0001).

#### 3.3.2. Targeting Bile Acid Metabolisms

##### FXR Agonists

Farnesoid X receptor (FXR), predominantly expressed in the liver and intestine, acts as a key regulator of bile acid homeostasis [182]. Its interaction with BAs not only negatively regulates BA synthesis but also suppresses hepatic adipogenesis and steatosis, reduces gluconeogenesis, and enhances peripheral insulin sensitivity, which leads to the remission of inflammation and fibrosis [183,184]. The expression of FXR in the liver is negatively associated with disease severity in patients with NASH [55]. Thus, FXR plays a pivotal role in maintaining metabolic and hepatic health, making it a crucial focus in therapeutic strategies.

Obeticholic acid (OCA), a 6α-ethyl derivate of chenodeoxycholic acid, represents the first FXR agonist and efficiently activates FXR [185,186]. The medicine was first approved by the FDA for marketing in May 2016 and was launched by the European Union a few months after that, being used as a second-line treatment for patients with primary biliary cholangitis (PBC) who are ineffective or intolerant to ursodeoxycholic acid [187]. As for the treatment of NASH, in a phase III REGENERATE study (NCT02548351), 23% of the patients in the 25 mg OCA dose group achieved at least a 1-stage improvement in fibrosis compared to 12% in the placebo group (*p* = 0.0002) at the pre-specified Month 18 interim analysis [188]. However, there were no significant improvements in NASH and the main side effects of OCA included pruritus and dyslipidemia [189]. However, due to safety concerns and the failure to meet both primary endpoints, the FDA declined to approve the new drug application for OCA.

In addition to OCA, there are also several non-steroidal FXR ligands without bile acid structures, such as cilofexor, tropifexor, and EDP-305, which may result in distinct pharmacokinetic properties, efficacies, and safety profiles. In a phase 2 trial, MRI-PDFF decreased by 22.7% after 24 weeks of treatment with 100 mg cilofexor compared to a 1.9% increase in the placebo group (*p* = 0.003), indicating that cilofexor was well tolerated over 24 weeks and significantly reduced hepatic steatosis, liver enzyme levels, and serum bile acids in NASH patient [190]. Currently, many studies focus on combination therapies including cilofexor for NASH treatment, offering the potential for fibrosis regression in patients with advanced fibrosis caused by NASH [191]. Tropifexor (LJN-452), a potent non-bile acid FXR agonist, significantly reduced ALT levels and liver fat content in a phase II trial (NCT02855164). Similarly to other FXR agonists, dose-dependent pruritus was a common side effect [192]. Another FXR agonist, EDP-305, is also under development. In its double-blind phase II study, patients receiving 2.5 mg EDP-305 showed a mean ALT reduction of −27.9 U/L compared to −15.4 U/L in the placebo group at week 12 [193]. However, nearly all the FXR agonists were associated with certain side effects, including pruritus and dyslipidemia.

#### 3.3.3. Targeting Inflammation and Fibrogenesis

##### ASK1 Inhibitors

Tumor necrosis factor receptor-associated factor 1 (TRAF1) is a key adapter protein involved in the regulation of immunity, inflammation, and cell death, which plays a pivotal role in promoting insulin resistance, inflammation, and hepatic steatosis through the activation of the ASK1-P38/JNK signaling pathway [194]. These functions prompted the development of selonsertib, an ASK1 inhibitor, which was evaluated in two phase III trials (STELLAR 3 and STELLAR 4) targeting patients with NASH and advanced fibrosis or cirrhosis despite demonstrating strong anti-inflammatory and antifibrotic effects in preclinical models [195,196].

##### CCR2/CCR5 Antagonists

The CCR2–CCR5 chemokine axis enhances the liver’s innate immune response, serving as a bridge between inflammation and the activation of hepatic stellate cells, which is an important driver of NASH [197]. Cenicriviroc (CVC) is a dual antagonist of the CCR2/CCR5 and is capable of inhibiting these receptors on macrophages and HSCs [198]. Although CVC demonstrated favorable tolerability and antifibrotic efficacy after one-year use in adults, the mean fibrosis stage showed no significant difference compared to those on placebo after two years [199,200]. A long-term phase III AURORA study (NCT03028740) was terminated early due to insufficient efficacy; however, CVC continues to be explored in combination therapy approaches [201].

##### Galectin 3 Inhibitors

Galectin 3 is a cytosolic protein secreted by macrophages in response to tissue damage, where it plays a key role in promoting fibrosis [202]. Belapectin, an inhibitor of galectin-3, has no significant effect on liver fibrosis or NASH activity score; however, it lowers HVPG and inhibits the progression of esophageal varices in patients without esophageal varices [203]. Therefore, a trial evaluating the safety and efficacy of belapectin in preventing esophageal varices in NASH cirrhosis is currently underway (NCT04365868). Another drug targeting the galectin-3 receptor, GB1211, is also in clinical trials. In the cohort of decompensated cirrhosis patients, GB1211 was reported to have good tolerability and reduced the levels of the liver injury-related markers ALT, AST, and GGT, indicating that the drug can be used for liver function impairment in the future [204].

##### LOXL-2 Inhibitors

Lysyl oxidase-like protein 2 (LOXL2), a member of the lysyl oxidase family, plays a critical role in the formation of cross-linked collagen and elastin in the extracellular matrix [205]. Recent studies have highlighted the therapeutic potential of LOXL2 inhibition in mouse models of liver fibrosis, suggesting that LOXL2 could be a promising target for treating hepatic fibrosis [206,207]. However, the antibody simtuzumab, designed to inhibit the collagen cross-linking activity of LOXL2, failed to show antifibrotic efficacy in clinical trials involving patients with advanced fibrosis or cirrhosis [208]. Recent research indicated that MSC-sEV^miR−4465^ effectively delivers miR-4465, reducing HSC activation and mitigating the progression of liver fibrosis by downregulating LOXL2. This modified MSC-sEV demonstrated significant potential in treating liver diseases and held considerable clinical promise [209].

##### FGF-21 Analogs

FGF21, also known as fibroblast growth factor 21, is a stress-inducible hormone produced in the liver and other metabolically active tissues [210,211]. FGF21 binds to fibroblast growth factor receptor (FGFR) 1c, 2c, and 3c and activates various intracellular signaling pathways to exert its biological effects. It plays a role in reducing liver stress, protecting liver cells, inhibiting cell apoptosis and inflammatory signals, and inhibiting the differentiation of hepatic stellate cells into collagen-secreting myofibroblasts [212]. Wu et al. found that the constitutive activation of HO-1 is a mediator of iron overload-induced ferroptosis in hepatocytes, and FGF21 may protect hepatocytes from this ferroptosis by promoting the ubiquitination and degradation of HO-1, which suggests that targeting the FGF21–HO-1 pathway could be a therapeutic approach for managing liver injury and fibrosis [213]. In 2022, Akero Therapeutics announced that its FGF-21 long-acting analog Efruxifemin (AKR-001) can improve liver fibrosis and prevent disease progression in patients with pre-cirrhosis NASH in phase IIb clinical trials [214]. After 24 weeks of treatment, both the primary and secondary endpoints showed statistical significance, with improved liver fibrosis and no deterioration of NASH. Another FGF-21 analog, pegozafermin, also achieved positive results in phase IIb clinical trials for the treatment of NASH patients. In NASH patients with moderate to severe liver fibrosis, subcutaneous injections of pegozafermin at doses of 15 mg or 30 mg weekly, or 44 mg every two weeks, led to fibrosis improvement without worsening NASH after 24 weeks [215].

##### TGF-β Inhibitors

Transforming growth factor-beta (TGF-β), a cytokine with immunosuppressive and profibrotic properties, plays a pivotal role in hepatic fibrosis by mediating the activation of stellate cells [216]. The expression of TGF-β1 is upregulated in both experimental hepatic fibrosis models and patients with liver fibrosis [217,218]. Galunisertib (LY2157299), an antifibrotic TGF-β inhibitor, demonstrated comparable antifibrotic potency in rat and human livers by inhibiting SMAD2 phosphorylation [219,220]. A novel synthetic verbenone derivative, SP-1154, significantly improved insulin sensitivity and glucose homeostasis, and reduced hepatic steatosis in the NAFLD mouse model [221]. Oxy210, an oxysterol derivative, exhibits antagonistic effects on Hedgehog (Hh) and TGF-β signaling in primary human HSCs. It effectively improved liver fibrosis and inflammation and alleviated hypercholesterolemia in a mice model, which indicated that it may serve as a potential therapeutic candidate for NASH [222]. Although the inhibition of TGF-β has shown promising results in animal models, its complex roles in cell proliferation, carcinogenesis, and immunity are currently the main issues for application in humans [223].

##### Phosphodiesterase Inhibitor

Pentoxifylline (PTX), a phosphodiesterase inhibitor primarily used in managing peripheral vascular diseases, has emerged as a promising therapeutic option for NAFLD/NASH. Its potential lies in its antioxidant properties and ability to suppress pro-inflammatory cytokines [224]. Additionally, PTX exhibits diverse pharmacological effects, including the inhibition of fibrosis-promoting factors like platelet-derived growth factor (PDGF) and TGF-β1 [225]. Zein et al. [226] reported that PTX significantly reduced steatosis, lobular inflammation, and liver fibrosis. Similarly, two meta-analyses highlighted PTX’s ability to lower liver enzyme levels and improve histological outcomes in NASH patients [227,228]. Hamouda et al. [229] demonstrated that PTX, alone or combined with KP, exerted significant ameliorative effects via mechanisms such as regulating apoptosis and necroptosis, reducing oxidative stress and lipogenesis, suppressing pro-inflammatory cytokines, and improving histopathological manifestations in NASH mice. Moreover, a prospective clinical study revealed that 24 weeks of PTX treatment led to a significant reduction in liver enzyme levels and improved insulin resistance in NASH patients [230]. In 2015, the Japanese Society of Gastroenterology (JSGE) recommended the use of PTX in patients with NASH. However, while PTX is considered a viable option for NAFLD, further high-quality, large-scale randomized controlled trials with refined inclusion criteria are essential to establish its efficacy and safety more definitively.

The drug targets are summarized in the following table (Table 1).

## 4. Novel Therapies

### 4.1. Cell Therapy

Cell-based therapies offer promising antifibrotic strategies for NASH, focusing on targeting HSCs and fibrogenic pathways [231]. Mesenchymal stromal cells (MSCs), derived from sources like umbilical cord, bone marrow, and adipose tissue, demonstrate therapeutic potential by reducing hepatocyte damage, inhibiting HSC activation, and promoting matrix metalloproteinase (MMP) synthesis, leading to fibrosis regression [232]. Umbilical cord-derived MSCs (UC-MSCs) have shown efficacy in improving liver histology and reducing fibrosis in animal models and clinical trials (NCT01220492) [233]. Their exosomes further provide cell-free benefits by modulating lipid metabolism and delivering antifibrotic miRNAs, such as miR-627-5p [234] and miR-146a-5p [235], to alleviate liver damage. Moreover, bone marrow-derived MSCs (BMSCs) contribute to fibrosis reduction by regulating macrophage phenotypes and pathways like Nrf2/HO-1 and Notch, suppressing inflammation, and improving mitochondrial function [236,237]. Another strategy, overexpressing CCR2 in MSCs, enhances targeted migration to damaged livers, improving therapeutic outcomes [238].

Macrophage is another potential option for cell therapy because of its ability to promote fibrosis regression and tissue regeneration. A phase I trial in compensated cirrhosis showed that autologous restorative macrophages derived from CD14^+^ monocytes were safe and slightly reduced liver disease severity [239]. However, challenges remain regarding engraftment efficiency, stability, and optimal cell sources, suggesting the potential of reprogrammed macrophages, such as chimeric antigen receptor (CAR)-expressing macrophages, for improved outcomes [240]. Furthermore, CAR-T cells, initially developed for hematological malignancies, show promise in treating liver fibrosis. Promising results have been shown in preclinical models by targeting fibrotic markers like uPAR and FAP, though challenges such as phenotype stability and off-target effects remain [241].

### 4.2. Genetic Approaches

Genetic factors play a significant role in NAFL/NASH susceptibility, and advanced mapping approaches offer the potential to identify diagnostic and therapeutic targets [242]. A splice variant (rs72613567:TA) in HSD17B13, identified in the DiscovEHR study, was associated with reduced ALT/AST levels and improved liver histology in NASH, offering protection against NASH and cirrhosis [243,244]. Despite promising findings in humans, HSD17B13-knockout mice failed to replicate these benefits, emphasizing species-specific considerations in drug development [245]. RNAi therapy targeting HSD17B13, such as ARO-HSD, has shown significant reductions in hepatic mRNA and ALT levels in early trials (NCT04202354) [246].

The PNPLA3-I148M variant, strongly linked to steatosis and fibrosis, highlights another genetic target. Silencing PNPLA3 using GalNAc-conjugated ASOs has demonstrated reductions in hepatic lipogenesis and steatosis in preclinical studies [247,248,249]. Clinical trials, such as ION839 (NCT04483947), are exploring its therapeutic potential. Additionally, ASOs targeting DGAT2 (IONIS-DGAT2Rx) and siRNAs like GalNAc-siTAZ show promise in reducing liver steatosis, inflammation, and fibrosis [250]. Emerging therapies targeting pathways like Notch using agents such as γ-secretase inhibitors [251] and Ncst ASO [252] further underscore the potential of genetic approaches to transform NAFL/NASH treatment. These advancements represent a promising frontier in combating liver disease through precision medicine.

### 4.3. Combination Therapies

NASH is a multifactorial disease involving intricate pathways of metabolism, inflammation, and fibrosis, making single-agent therapies insufficient to address its complexity. Combination therapies have emerged as a promising strategy to target multiple pathogenic mechanisms simultaneously, enhancing efficacy while minimizing dose-dependent side effects [253]. The European Medicines Agency recommends using agents with complementary mechanisms of action. For instance, the combination of cilofexor (FXR agonist) and firsocostat (ACC inhibitor) demonstrated superior efficacy in reducing hepatic fat and fibrosis while limiting complications like pruritus and LDL elevation compared to cilofexor alone (NCT03449446) [191].

Other trials have explored combinations such as tropifexor (non-bile acid FXR agonist) with cenicriviroc (CCR2/5 antagonist), aiming to synergistically address steatosis, inflammation, and fibrosis (NCT03517540) [254]. Preclinical studies also highlight the potential of multi-target agents, like ZLY18 (a quadruple FFA1/PPAR-α/γ/δ agonist), which significantly improved liver histology in NASH models, warranting further investigation [255]. Meanwhile, the ATLAS trial revealed that pairing firsocostat, cilofexor, and selonsertib outperformed individual agents in fibrosis reduction but showed varying efficacies across combinations (NCT03449446) [191]. Additionally, semaglutide, an antidiabetic GLP1 receptor agonist, combined with cilofexor and firsocostat, demonstrated enhanced benefits over monotherapy, reflecting the potential of cross-class combinations [256].

Despite encouraging early results, challenges such as optimal combination selection, side effect management, and treatment duration persist. Long-term follow-up in ongoing phase III and IV trials will be critical to establishing whether histological improvements translate into clinical benefits. The prospect of using personalized biomarkers to tailor combination therapies offers hope for maximizing efficacy and cost-effectiveness while addressing the chronicity of NASH [257].

## 5. Discussion

**Figure 3:**
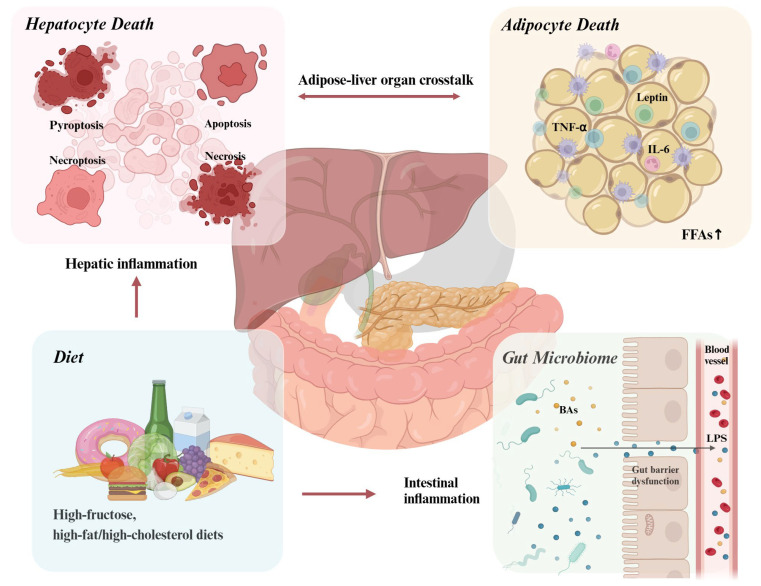
Triggers of the inflammation in NAFLD. Triggers of inflammation in NAFLD involve complex interactions among metabolic, cellular, and microbial factors that drive liver injury and disease progression. Adipocyte death releases cytokines, lipotoxic substances, and free fatty acids, worsening liver injury and fibrosis, while hepatocyte death activates inflammatory pathways and fibrosis via DAMPs. Gut microbiota dysbiosis disrupts the gut–liver axis, allowing endotoxins like LPS to trigger hepatic inflammation through Toll-like receptors. High-fructose and high-fat diets exacerbate oxidative stress, lipotoxicity, and mitochondrial dysfunction, intensifying liver inflammation and metabolic imbalance. (Created in https://BioRender.com). The red arrows represent the interactions between different components, while the black arrows indicate substances (such as LPS) entering the bloodstream from the intestine.

Inflammation plays a critical role in the progression of NAFLD to NASH. Mechanisms such as adipocyte dysfunction, lipotoxicity, and gut microbiota dysbiosis contribute to liver injury and fibrosis by activating inflammatory pathways (Figure 3). Pro-inflammatory cytokines like TNF-α and IL-6 drive hepatic damage, while protective adipokines such as adiponectin are often reduced, further exacerbating disease progression. Dysregulated gut microbiota promotes gut–liver axis dysfunction, enabling bacterial endotoxins to enter the liver and trigger inflammation. Therapeutic strategies targeting these inflammatory processes have shown promise in preclinical studies and early clinical trials, but their application in routine clinical practice remains limited due to mixed efficacy and concerns about long-term safety.

Lifestyle interventions, such as diet and exercise, form the cornerstone of NAFLD/NASH management, yet they are often insufficient for advanced disease. Pharmacological agents, including vitamin E, SGLT-2 inhibitors, and PPAR agonists, have shown potential in addressing specific aspects of NASH but fail to comprehensively resolve inflammation and fibrosis. Moreover, their utility is frequently limited by side effects and variability in patient response, reflecting the multifaceted nature of the disease.

Emerging therapies aim to overcome these limitations by addressing multiple pathogenic pathways simultaneously. Approaches such as incretin-based agents, bile acid modulators, and genetic therapies offer promise, while combination treatments are increasingly recognized for their ability to target inflammation, metabolic dysfunction, and fibrosis concurrently. These strategies signify a shift toward more holistic and personalized treatment paradigms. However, achieving widespread efficacy and safety remains a significant hurdle, highlighting the complexity of NASH pathogenesis and the need for continued innovation.

## 6. Conclusions

NAFLD and NASH are complex metabolic liver diseases with significant global health implications. Inflammation is a key driver of disease progression, making it an essential focus for therapeutic intervention. The current treatments, including lifestyle modifications and pharmacological agents, offer partial benefits but remain inadequate for managing advanced disease stages. The multifactorial nature of NASH underscores the need for integrative approaches targeting inflammation, metabolic dysfunction, and fibrosis. Continued advancements in understanding disease mechanisms and developing comprehensive treatment strategies are critical to addressing this growing health challenge effectively.

## 7. Future Directions

The future of NASH treatment hinges on a multifaceted approach that addresses the disease’s complexity and heterogeneity. Combination therapies are likely to be central, targeting inflammation, fibrosis, and metabolic dysfunction simultaneously. However, given individual variability, tailored strategies—such as remission induction, maintenance protocols, or periodic therapeutic cycles—will be essential to optimize outcomes. Moreover, advancements in precision medicine will be key to overcoming the current therapeutic challenges. By integrating genomic, phenomic, and transcriptomic data, researchers can identify specific disease drivers and design targeted therapies. Tools like polygenic risk scores could guide individualized treatment, while non-invasive diagnostics, such as circulating free DNA, could enable earlier and safer detection, reducing reliance on liver biopsies.

In parallel, the search for novel therapies continues, with RNA-based drugs and other genetic interventions, such as those targeting PNPLA3 and HSD17B13, showing early promise. Furthermore, innovative preclinical models, including liver organoids and humanized mice, are poised to bridge the gap between experimental and clinical settings, enabling more precise drug development.

Ultimately, the future of NASH treatment will likely rely on integrating pharmacological therapies with lifestyle interventions, using combination strategies to address the disease comprehensively. With ongoing research, technological advancements, and international collaboration, there is hope for safer, more effective treatments that can significantly improve patient outcomes.

## 8. Methods

This review was conducted to summarize the current understanding of inflammation in the progression of NAFLD/NASH, and to evaluate therapeutic targets and approaches to manage inflammation. A comprehensive literature search was performed using the following databases: PubMed, Web of Science, and Scopus. The search covered studies published until June 2024, ensuring the inclusion of the most recent and relevant research.

The keywords used for the search included “NAFLD”, “NASH”, “inflammation”, “pathophysiology”, “therapeutic targets”, “drug development”, “fibrosis”, and “metabolic dysfunction”. Boolean operators (e.g., AND and OR) were applied to combine these terms effectively. Both original research articles and review papers were included, with a preference for peer-reviewed and high-impact publications. Additionally, guidelines and consensus statements from authoritative organizations were referenced to ensure the inclusion of widely accepted perspectives.

The articles were selected based on their relevance to the topic, with an emphasis on the studies exploring the pathophysiological mechanisms of inflammation, potential therapeutic targets, and clinical trials focusing on drug development for NAFLD/NASH. Non-English publications, case reports, and studies lacking full text were excluded.

This methodology ensured a comprehensive and balanced assessment of the literature, allowing for a critical discussion of the current progress, challenges, and future directions in managing inflammation in NAFLD/NASH.

## Figures and Tables

**Table 1:** Drugs in clinical trials for NAFLD and NASH treatment.

Class	Drug	Mechanism	Cohort	NCT Number	Reference
AMPK activator	Metformin	Reduces glucose production in hepatocytes and improves insulin resistance	T2DM patients with NASH (fibrosis or cirrhosis)	NCT00063232	[121]
	Ipragliflozin	Reduces glucose reabsorption in the renal proximal tubules	NAFLD patients with diabetes	UMIN000015727 UMIN 000022651	[127]
SGLT-2 inhibitors	Empagliflozin	Reduces steatosis, ballooning, and fibrosis	NAFLD patients with T2DM	NCT04642261	[128,129]
	Canagliflozin	Reduces hepatic steatosis and insulin resistance	T2DM patients with chronic kidney disease	UMIN000023044 UMIN000020615	[130]
PPAR agonist	Dapagliflozin	Reduces steatosis and fibrosis	T2DM patients with NAFLD	NCT03723252	[131]
	Pemafibrate	PPARα agonist	Patients with NAFLD	NCT03350165	[135]
	Pioglitazone	PPARγ agonist	Patients with NASH with or without T2DM	NCT00994682	[139]
	Seladelpar	PPARδ agonist	Patients with NASH	NCT03551522(terminated)	[141]
	Saroglitazar	Dual PPARα/γ agonist	Patients with NAFLD	NCT03061721	[142]
	Elafibranor	Dual PPARα/δ agonist	Patients with NASH	NCT01694849	[143]
	Lanifibranor	Pan-PPAR agonist	Patients with NASH	NCT03008070	[145]
GLP1R/GCGR/GIPR agonists	Semaglutide	GLP1R agonist; increase insulin secretion and sensitivity	Patients with NASH	NCT02970942 NCT04822181	[149]
	Efinopegdutide	GLP1R/GCGR dual agonist	Patients with NAFLD	NCT04944992	[150]
	Cotadutide	GLP1R/GCGR dual agonist	Patients with T2DM	NCT03555994	[151]
	Pemvidutide	GLP1R/GCGR dual agonist	Obesity patients with NAFLD	NCT05006885	[152]
	Survodutide	GLP1R/GCGR dual agonist	Patients with NASH and fibrosis stage F1-F3	NCT04771273	[153]
	Tirzepatide	GLP1R/GIPR dual agonist	Patients with NASH	NCT03131687 NCT04166773	[154]
	Retatrutide	GCGR/GIPR/GLP1R agonist	Obesity adults	NCT04881760	[155]
HMG-CoA reductase inhibitors	Rosuvastatin	Reduces the risk of HCC development	Patients with NAFLD	NCT03434613	[158,159]
NPC1L1 inhibitor	Ezetimibe	Reduces cholesterol absorption	Patients with NAFLD	NCT03434613	[164]
ACC inhibitor	GS-0976	Inhibits the conversion from acetyl-CoA to malonyl-CoA; suppresses DNL	Patients with NAFLD	NCT02856555	[165,166]
	MK-4074	Reduces hepatic steatosis	Patients with NAFLD	NCT01431521	[167]
SCD1 inhibitor	Aramchol	Inhibits the conversion from stearoyl-CoA and palmitoyl-CoA	Patients with NASH	NCT02279524 NCT04104321	[175]
THR-β agonists	Resmetirom	Activates THR-β in the liver selectively; promotes liver fat decomposition	Patients with NASH (with liver fibrosis)	NCT02912260 NCT03900429 NCT04197479	[179,180]
	VK2809	Reduces plasma and liver lipids; improves liver fibrosis	Patients with NAFLD	NCT02927184	[181]
FXR agonists	Obeticholic acid	Increases insulin sensitivity; improves hepatocyte cytotoxicity	Patients with NASH	NCT02548351	[188]
	Cilofexor	Reduces liver fibrosis and intrahepatic sinusoidal resistance; inhibits fibrogenesis	Patients With Noncirrhotic NASH	NCT03890120	[190]
	Tropifexor	Reduces lipid synthesis; improves fibrosis and steatosis	Patients with NASH	NCT02855164 NCT03421431	[192]
	EDP-305	Inhibits lipogenesis; improves inflammation and steatosis	Patients with NASH	NCT04378010	[193]
ASK1 inhibitors	Selonsertib	Inhibits the formation of inflammatory cytokines	Patients with NASH and advanced fibrosis or cirrhosis	NCT03053050	[195,196]
CCR2/CCR5 inhibitors	Cenicriviroc	Inhibits both CCR2 and CCR5; improves inflammation and fibrosis.	Adults with NASH and Fibrosis	NCT03028740	[199,200]
Galectin 3 antagonist	Belapectin	Reduces HVPG and development of varices	Patients with NASH with cirrhosis and Portal Hypertension	NCT04365868	[203]
FGF-21 analogs	Efruxifemin	Stimulates the agonisms of FGFR1c, FGFR2c, and FGFR3c	Patients with NASH	NCT04767529 NCT04929483	[214]
	Pegozafermin	Stimulates FGF21; improves insulin resistance	Patients with pre-cirrhosis NASH	NCT04048135	[215]
Phosphodiesterase inhibitor	Pentoxifylline	Exhibits antioxidant activity and suppresses pro-inflammatory cytokines	Patients with NASH	NCT00590161	[226]

## Data Availability

Data sharing is not applicable.

## Abbreviations

The following abbreviations are used in this manuscript:

NAFLD	non-alcoholic fatty liver disease
NASH	non-alcoholic steatohepatitis
HCC	hepatocellular carcinoma
SAF	Steatosis activity fibrosis
NAS	NAFLD activity score
MASLD	metabolic dysfunction-associated steatotic liver disease
IR	insulin resistance
TNF-α	Tumor necrosis factor-α
IL-6	interleukin-6
HSC	hepatic stellate cell
ER	endoplasmic reticulum
DAMP	damage-associated molecular patterns
RIPK3	Receptor-interacting serine/threonine-protein kinase 3
GSDMD	Gasdermin D
SMS1	Sphingomyelin synthase 1
LPS	lipopolysaccharide
TLR	Toll-like receptor
FXR	farnesoid X receptor
TGR5	G protein-coupled bile acid receptor 1
DNL	de novo lipogenesis
NOX2	NADPH oxidase 2
UPR	unfolded protein response
PUFAs	polyunsaturated fatty acids
RCTs	randomized controlled trials
BMI	body mass index
FMT	fecal microbiota transplantation
ALT	alanine aminotransferase
AST	Aspartate aminotransferase
PBC	primary biliary cholangitis
TRAF1	Tumor necrosis factor receptor-associated factor 1
ASK1	Apoptosis signal-regulating kinase 1
CCR2/CCR5	C-C motif chemokine receptor 2/5
HVPG	Hepatic venous pressure gradient
FGF-21	fibroblast growth factor 21
MSC	mesenchymal stromal cells
BMSCs	bone marrow-derived mesenchymal stromal cells
CAR-T	chimeric antigen receptor T cell
ASOs	Antisense oligonucleotides
AMPK	AMP-activated protein kinase
ASCA	Anti-saccharomyces cerevisiae antibodies
NEUT%	Neutrophil percentage
HGB	Hemoglobin
PLT	platelet
Alb	Albumin
TBil	Total bilirubin
Cr	Creatinine
T-25OHD	25-Hydroxyvitamin D
Fe	Serum iron
TS	Transferrin saturation
TIBC	Total iron-binding capacity
hsCRP	High-sensitivity c-reactive protein
ESR	Erythrocyte sedimentation rate
CMV DNA	Cytomegalovirus DNA
EBV DNA	Epstein–Barr virus DNA
WBC	White blood cell count
LDL	Low-density lipoprotein
FGFR	fibroblast growth factor receptor
ACC	acetyl-CoA carboxylase
SCD1	stearoyl-CoA desaturase 1
THR-β	Thyroid hormone receptor beta
NPC1L1	Niemann-pick C1-like 1
GLP1R	glucagon-like peptide-1 receptor
GCGR	glucagon receptor
GIPR	glucose-dependent insulinotropic polypeptide receptor
T2DM	type 2 diabetes mellitus
EMA	European Medicines Agency
FDA	Food and Drug Administration
MRI-PDFF	Magnetic resonance imaging proton density fat fraction
PRO-C3	Procollagen type III C-peptide
MMP	matrix metalloproteinase
TGF-β	Transforming growth factor-beta
PTX	Pentoxifylline
PDGF	platelet-derived growth factor
KP	Ketogenic peptide
JSGE	Japanese Society of Gastroenterology
LOXL2	Lysyl oxidase-like protein 2
HSD17B13	Hydroxysteroid 17-beta dehydrogenase 13
PNPLA3	Patatin-like phospholipase domain-containing protein 3
DGAT2	Diacylglycerol O-Acyltransferase 2
